# New isotope constraints on the Mg oceanic budget point to cryptic modern dolomite formation

**DOI:** 10.1038/s41467-019-13514-6

**Published:** 2019-12-11

**Authors:** Netta Shalev, Tomaso R. R. Bontognali, C. Geoffrey Wheat, Derek Vance

**Affiliations:** 1 Department of Earth Sciences, Institute of Geochemistry and Petrology, ETH Zürich, Clausiusstrasse 25, 8092 Zürich, Switzerland; 2 Space Exploration Institute, Fbg de l’Hopital 68, 2002 Neuchâtel, Switzerland; 3 Department of Environmental Sciences, University of Basel, Klingelbergstrasse 27, Basel, Switzerland; 4 University of Alaska Fairbanks, PO Box 475, Moss Landing, California 95039 USA

**Keywords:** Element cycles, Palaeoceanography, Marine chemistry

## Abstract

The oceanic magnesium budget is important to our understanding of Earth’s carbon cycle, because similar processes control both (e.g., weathering, volcanism, and carbonate precipitation). However, dolomite sedimentation and low-temperature hydrothermal circulation remain enigmatic oceanic Mg sinks. In recent years, magnesium isotopes (δ^26^Mg) have provided new constraints on the Mg cycle, but the lack of data for the low-temperature hydrothermal isotope fractionation has hindered this approach. Here we present new δ^26^Mg data for low-temperature hydrothermal fluids, demonstrating preferential ^26^Mg incorporation into the oceanic crust, on average by *ε*_solid-fluid_ ≈ 1.6‰. These new data, along with the constant seawater δ^26^Mg over the past ~20 Myr, require a significant dolomitic sink (estimated to be 1.5–2.9 Tmol yr^−1^; 40–60% of the oceanic Mg outputs). This estimate argues strongly against the conventional view that dolomite formation has been negligible in the Neogene and points to the existence of significant hidden dolomite formation.

## Abstract (web summary)

Earth’s carbon cycle and oceanic magnesium cycle are controlled by processes such as weathering, volcanism and precipitation of carbonates, such as dolomite. Here, the authors contradict the view that modern dolomite formation is rare and suggest instead that dolomite accounts for ~40–60% of the global oceanic Mg output in the last 20 Ma.

## Introduction

**Fig. 1:**
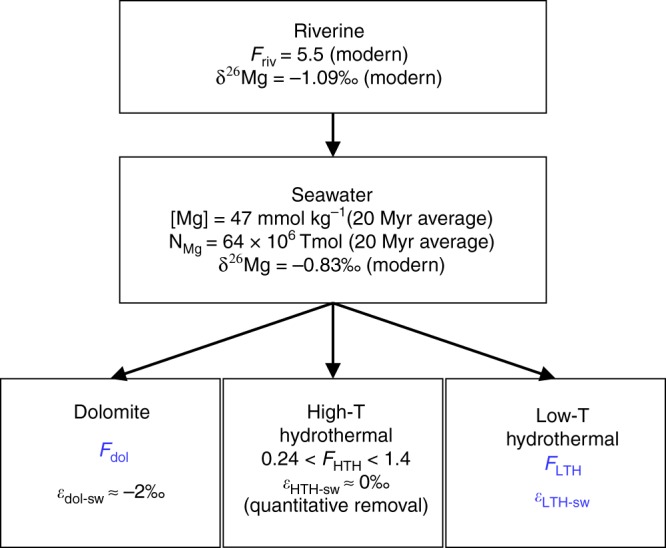
Schematic of the oceanic Mg budget. Modified after: Tipper et al.^15^. In blue are the missing parameters determined in this study. Mg input or output fluxes, *F*, are in Tmol yr^−1^ and are taken from the following: Berner and Berner^16^, Elderfield and Schultz^6^, Arvidson et al.^3^, and Vance et al.^17^ and references therein. The subscripts riv, dol, HTH, LTH, and sw stand for riverine, dolomite, high-temperature hydrothermal, low-temperature hydrothermal, and seawater, respectively. *N*_Mg_ is the Mg inventory in seawater. δ^26^Mg values are taken from Tipper et al.^15^, Foster et al.^18^, and Ling et al.^19^. *ε*_sink-sw_ is the isotope fractionation between seawater and the specified Mg sink. *ε*_dol-sw_ is taken from Higgins and Schrag^20^, Li et al.^21^, and others. Only the major oceanic inputs and outputs are shown. For details see text.

Magnesium is mainly supplied to the oceanic dissolved pool by chemical weathering and the transport of its products in rivers (Fig. 1)^e.g., 1–3^. It is removed from the oceans mainly by the formation of Mg-rich carbonates (mostly dolomite)^e.g., 2,4,5^ and by hydrothermal reactions within the oceanic crust (Fig. 1)^e.g., 2,3,6^. Such reactions occur at high temperatures (≥70 °C) at mid-ocean ridges (high-temperature hydrothermal, HTH), where hydrothermal systems are driven by heat derived from the intrusion of magma, and at low temperatures (<70 °C; low-temperature hydrothermal, LTH) on mid-ocean ridge flanks, where hydrothermal systems are driven by conductive cooling of the crust^e.g., 7,8^. The same mechanisms (i.e., weathering, volcanism, and carbonate precipitation) also control the atmospheric inventory of CO_2_. Thus, the Mg budget of the ocean is fundamentally linked to that of carbon and therefore with long-term climate change^e.g., 9^. However, removal fluxes of Mg via the three major processes mentioned above (dolomite formation, HTH, and LTH; Fig. 1) are poorly constrained, hindering the use of the history of Mg in seawater to understand processes that affect long-term climate change and the proxies used to study it. In particular, a ~60% rise in seawater Mg concentration has been observed during the Cenozoic, along with a factor of 2–3 rise in Mg/Ca ratio. The precise driving mechanism is highly contested, but has important implications for our understanding of global carbon cycling during this time interval^e.g., 10–14^.

Uncertainties in the oceanic Mg budget are exacerbated by debate over the recent (since the beginning of the Neogene) dolomite Mg-removal flux, with estimates varying by more than an order of magnitude^e.g., 2,3^. According to the conventional view, dolomite formation mainly occurs on continental shelves, which are limited in the modern ocean^e.g., 22–24^. Therefore, the modern dolomite Mg flux is regarded as negligible (~0.1 Tmol yr^−1^)^3,5,22^. On the other hand, Holland^2^ suggests a modern Mg flux of ~1.7 Tmol yr^−1^, similar to the average for the Phanerozoic (1.8 Tmol yr^−1^)^25^.

**Fig. 2:**
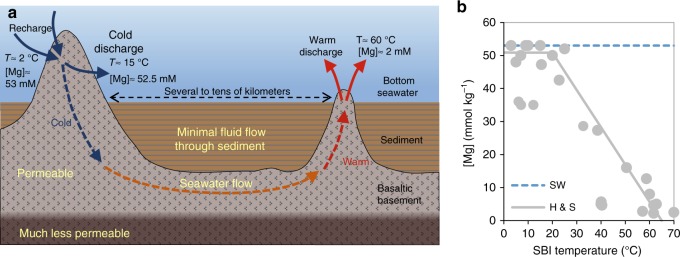
The low-temperature hydrothermal (LTH) system. **a** Schematic illustration of ridge-flank hydrothermal circulation and its effect on temperature, T, and Mg concentration, [Mg] (after Fisher and Wheat^29^). The sediment cover has low permeability relative to the basalt that comprises the upper permeable portion of the basaltic basement^35^. **b** Concentration of Mg vs. temperature at the sediment-basement-interface (SBI) in LTH fluids (data from Wheat et al.^34,36,37,39^, Fisher et al.^38^, and Fisher and Wheat^29^ and references therein). Blue-dashed line (SW) is seawater Mg concentration (~53 mmol kg^−1^). Gray-solid line (H&S) is a modeled temperature dependence taken from Higgins and Schrag^20^.

Although the Mg flux into the HTH system (~0.2–1.4 Tmol yr^−1^) is better constrained by the water flux through mid-ocean ridge basalts (MORBs; ~0.5–3∙10^13^ kg yr^−1^)^26–28^ and by quantitative Mg removal at high temperatures^e.g., 6^, the removal of Mg at low temperatures on ridge flanks is more enigmatic (Fig. 2a)^e.g., 8^. The temperature of the upper oceanic crust is determined, in part, by the hydrology of the LTH system, i.e., by the flux of circulating seawater^e.g., 29^. Therefore, seawater fluxes through the global LTH system can be calculated from heat-loss considerations, assuming a global average temperature difference between bottom seawater and the upper crust (at the sediment–basement interface, SBI). Thus, Fisher and Wheat^29^ suggested a global LTH seawater flux of 10^15^–10^16^ kg yr^−1^ for temperature differences between 5 °C and 60 °C. Further, Mg removal at temperatures <65 °C is not quantitative and varies as a function of temperature (Fig. 2b)^8,29–31^. This further complicates the estimation of the global Mg flux into the LTH sink. Geophysical constraints suggest that most of the heat flow in the LTH system occurs at cold temperatures (<20 °C)^32,33^, where Mg removal is minimal. However, as the water fluxes are large, LTH systems are crucial for global Mg budgets^34^.

Exploring the Mg stable isotope compositions (δ^26^Mg = [(^26^Mg/^24^Mg)_sample_/(^26^Mg/^24^Mg)_DSM3_ −1]∙1000; see Methods for details) of seawater and oceanic Mg inputs and outputs can help constrain these fluxes, and ultimately their impact on the global ocean Mg budget and its evolution through time in response to changes in global exogenic cycles. Recent reconstructions of seawater δ^26^Mg indicate that the Cenozoic increase in Mg concentration is accompanied by minor isotopic changes^40,41^. However, the use of these records to quantify oceanic Mg input and output fluxes, and the implications for secular changes, depends fundamentally on a full understanding of Mg isotope cycles. The δ^26^Mg of modern seawater (−0.83‰)^18,19^ is higher than riverine sources (−1.09‰)^15^, thereby requiring an “isotopically light” sink (Fig. 1). Previous studies on dolomite formation in deep-sea pore-water and in shallow-water carbonate sediments indicate an isotope fractionation of *ε*_dol-sw_ ≈ −2‰^e.g., 20,21,42^ (see Methods for *ε* definition). The removal of Mg into the HTH sink is quantitative and therefore must be associated with no isotope effect. In contrast, Mg removal at LTH systems is partial (Fig. 2b) and will have a significant impact on the δ^26^Mg value of seawater. However, this impact has never been measured before.

Here we present the first δ^26^Mg data in LTH fluids, using samples that cover a wide range of SBI temperatures (6 °C – 64 °C) and Mg concentrations (1.3–52.5 mmol kg^−1^) from the Juan de Fuca plate (Ocean Drilling Program, ODP, sites 1024 and 1025; Integrated Ocean Drilling Program, IODP, sites 1362A/B and 1363B/D/F/G) and the eastern flank of the East Pacific Rise, Cocos plate (AT26-24 4775/4777; Supplementary Table 1). We document temperature-dependent preferential removal of heavy isotopes into the oceanic crust with a globally averaged fractionation, *ε*_LTH-sw_, of ~1.6‰. Finally, we extrapolate our results to constrain the oceanic Mg and Mg isotope budget over the past ~20 Myr and the implications for past variations in ocean chemistry and sedimentation patterns. This budget calculation suggests a significant LTH Mg sink, which requires a significant Mg sink to dolomite, to balance the Mg isotope budget. This suggested sink is larger than previously suggested, contrasting with the conventional view that dolomite formation has been negligible in the Neogene. Therefore, we suggest that models that link the apparent lack of dolomite in modern sediments to lower atmospheric CO_2_—lower sea levels—cooler climatic conditions need to be revised.

## Results

### δ^26^Mg values of low-temperature hydrothermal fluids

The studied samples are either crustal upper-basement fluids, from springs (AT26-09 and AT26-24 4775/4777; Dorado outcrop, Cocos Plate, Eastern flank of the East Pacific Rise) or borehole observatories (IODP Holes 1362A and 1362B; ODP Sites 1024 and 1025; Eastern flank of Juan de Fuca ridge), or sediment pore waters (IODP Sites U1363A-F; Grizzly Bare outcrop, Juan de Fuca plate). Details of the samples and their setting, and sampling methods, are summarized in the Methods section and in Supplementary Table 1, while further details can be found in Wheat et al.^34,36–39^. Pore waters do not necessarily represent the composition of the underlying basaltic basement fluids, as they may be affected by sediment diagenetic processes and diffusive fluxes from the overlying seawater. Therefore, where applicable, pore-water data for each site were depth-extrapolated to give the estimated value at the SBI^39^ (see Methods section for more details). Wheat et al.^37^ validated this approach by comparing the concentrations of various elements in basement fluid from Hole 1025 with the concentration calculated by extrapolating pore-water compositions to the SBI. They found that, for most elements (including Mg), the difference between these two methods was <5%. These extrapolated data are referred to hereafter as extrapolated SBI.

**Fig. 3:**
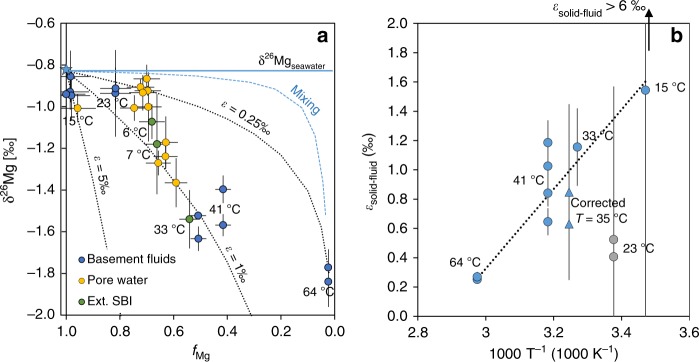
Mg isotopes in the low-temperature hydrothermal (LTH) system. **a** δ^26^Mg values of LTH fluids vs. the remaining fraction of Mg relative to bottom seawater, *f*_Mg_ (Table 1). Types of fluids are indicated by blue (basement fluids), yellow (sediments pore water), and green (extrapolated sediment-basement interface, Ext. SBI) circles. The pore-water sample from Site 1363G (see Supplementary Table 1) was collected right at the SBI and it is thus shown in green. Labels indicate the SBI temperature at each site. Blue-dashed line shows the trajectory expected for mixing between seawater and the warmest LTH endmember. Black-dotted lines show theoretical Rayleigh fractionation curves (see Methods) using different enrichment factors (*ε*_solid-fluid_). Modern seawater is indicated by a blue star and, for convenience, its δ^26^Mg value is shown by a blue solid line. Error bars are 2 SD. **b** The isotope fractionation, *ε*_solid-fluid_, vs. 1000 T^−1^, where *T* is the temperature at SBI in K (see also Table 1). Temperatures in °C are shown as labels. Two extremely high *ε*_solid-fluid_ are not shown (arrow). Triangles indicate that the SBI temperature was corrected to 35 °C, as suggested by the Mg concentrations (Holes 1363F and 1363G). Gray circles are the samples from Site 1024, which were excluded from the regression line. Analytical errors (2 SD) on the x-axis are within symbols. Error bars on y-axis are propagated 2 SD errors (Table 1). The black-dotted line is a linear regression through all data weighted by the error of each data point.

Mg isotope composition (δ^26^Mg) results are presented in Table 1 and Fig. 3a as a function of *f*_Mg_ (the remaining fraction of Mg relative to bottom seawater), to examine potential isotope fractionation mechanisms. The δ^26^Mg values of all samples are lower than the modern seawater value (−0.83‰; Table 1 and Fig. 3a), signaling preferential removal of heavy isotopes into the oceanic crust. This LTH isotope fractionation (positive *ε*_LTH-sw_) is in accordance with the higher δ^26^Mg values of the ~170 Myr-old oceanic crust samples recovered from the Western Pacific^43^ relative to fresh MORBs^44^ and it is in the opposite direction compared with the fractionation during dolomite formation (negative *ε*_dol-sw_). The warmest fluid (64 °C) has the lowest Mg concentration (1.3 mmol kg^−1^) and the most ^26^Mg-depleted isotopic composition (δ^26^Mg = −1.84 ± 0.12‰, 2 SD). In general, this depletion in ^26^Mg tends to decrease with increasing Mg concentrations and decreasing temperature, toward the values of bottom seawater. The observed relationships between temperature, Mg abundance, and δ^26^Mg could be interpreted as a result of mixing seawater and hydrothermal end members. However, a comparison with a simple mixing trend with the warmest endmember (from Site 1362; blue-dashed line in Fig. 3a) demonstrates that all samples are typically more depleted in ^26^Mg than would be expected in this case. Instead, the results imply that Mg removal at low temperatures is non-quantitative and fractionates Mg isotopes with variable positive enrichment factors (*ε*_solid-fluid_; black-dotted lines in Fig. 3a).

**Table 1:** Mg isotope values of the LTH fluids.

Sample	Temp. at SBI^a^ (°C)	Mg^a^ (mmol kg^−1^)	*f*_Mg_^b^	δ^26^Mg (‰)	2SD (‰)	δ^25^Mg (‰)	2SD (‰)	*n*	*ε*_solid-fluid_^c^ (‰)	*ε*-error^c^ (‰)
Basement (formation) fluids										
1362A-MVBS-13	64	1.3	0.02	−1.77	0.09	−0.94	0.11	4	0.25	0.02
1362B-sled #5	64	1.3	0.02	−1.84	0.12	−1.01	0.14	4	0.27	0.03
1024–46	23	43.2	0.82	−0.94	0.21	−0.53	0.22	4	0.52	1.04
1024-90	23	43.2	0.82	−0.91	0.10	−0.48	0.11	3	0.41	0.51
1025-#3-acidified	41	22.0	0.42	−1.40	0.07	−0.75	0.07	3	0.65	0.09
1025-#2-unacidified	41	43.4	NA	−0.95	0.05	−0.49	0.07	4	NA	NA
Corr.−1025-#2-unacidified^d^	41	22.0	0.42	−1.57	0.05	−0.83	0.06	NA	0.84	0.09
1025-3608-blue	41	26.9	0.51	−1.52	0.01	−0.78	0.03	4	1.03	0.11
1025-3608-red	41	27.7	NA	−1.58	0.09	−0.79	0.02	4	NA	NA
Corr.−1025-3608-red^d^	41	26.9	0.51	−1.63	0.06	−0.82	0.03	NA	1.19	0.15
Springs fluids										
4775-6-Dorado	15	52.5	0.99	−0.93	0.08	−0.54	0.14	6	7	33
4777-7-Dorado	15	52.4	0.98	−0.86	0.12	−0.45	0.16	5	1.5	10
4775-9-dorado	15	52.3	0.98	−0.95	0.12	−0.51	0.10	4	6	23
4777-6-Dorado	15	53.3	1.00	−0.94	0.17	−0.47	0.18	4	NA	NA
Pore water										
1363G-3H1	6.1^e^	36	0.68	−1.07	0.08	−0.62	0.11	6	0.63	0.25
1363D-4X1	ND	33.3	0.63	−1.17	0.13	−0.60	0.12	8	NA	NA
1363D-4X2	ND	33.3	0.63	−1.24	0.06	−0.66	0.04	6	NA	NA
1363D-4X3	ND	34.7	0.66	−1.27	0.06	−0.70	0.04	4	NA	NA
1363D-5X1	ND	31.3	0.59	−1.36	0.12	−0.72	0.10	8	NA	NA
1363D SBI-extrapolated^f^	32.7	28.6	0.54	−1.54	0.14	−0.83	0.21	4	1.16	0.26
1363B-4H5	ND	50.6	0.96	−1.01	0.07	−0.52	0.03	4	NA	NA
1363B-7X1	ND	39.5	0.75	−1.01	0.06	−0.51	0.02	4	NA	NA
1363B-8X2	ND	36.8	0.70	−0.92	0.02	−0.48	0.05	4	NA	NA
1363B-8X3	ND	38.2	0.72	−0.91	0.04	−0.49	0.04	6	NA	NA
1363B SBI-extrapolated^f^	12.2^e^	35	0.66	NA	NA	NA	NA	NA	NA	NA
1363F 4H1	ND	37.0	0.70	−0.87	0.07	−0.38	0.07	4	NA	NA
1363F 4H2	ND	37.8	0.71	−0.93	0.08	−0.46	0.08	4	NA	NA
1363F 4H3 IW29	ND	36.7	0.69	−1.00	0.17	−0.54	0.20	3	NA	NA
1363F SBI-extrapolated^f^	7.1^e^	35	0.66	−1.18	0.24	−0.75	0.27	3	0.85	0.60

*NA* not applicable, *ND* not determined

^a^SBI temperatures and Mg concentration data from Wheat et al.^34,36,37,39^, Fisher et al.^38^, and this study (see Supplementary Table 1)

^b^*f*_Mg_ is the remaining fraction of Mg relative to bottom seawater, which were taken as 52.9 mmol kg^−1^ in most cases. For Dorado samples, bottom seawater was taken as 53.3 mmol kg^−1^, the highest concentration measured there

^c^*ε*_solid-fluid_ was calculated assuming a Rayleigh distillation (see Methods), initial seawater δ^26^Mg = −0.83‰ and the measured *f*_Mg_ and δ^26^Mg of each sample. The error on the *ε* was propagated

^d^Corrected values for mixing with seawater are indicated by Corr

^e^Mg concentrations suggest that the temperature at SBI is 30–40 °C

^f^Pore water values were extrapolated to the SBI depth (see Supplementary Table 1 for temperature and Mg concentration data and references). δ^26^Mg values of pore water from 1363B show small variations and opposite correlation with depth and therefore an extrapolated value was not calculated

The variability in isotope fractionation between the basaltic host and circulating seawater (*ε*_solid-fluid_) can be assessed in the framework of a Rayleigh distillation model (see Methods for details) to relate the remaining fraction of Mg relative to bottom seawater, *f*_Mg_, and measured δ^26^Mg values, for each sample. In general, the isotope fractionation (*ε*_solid-fluid_) increases with decreasing SBI temperature, from ~0.25‰ at 64 °C to 1.5–7‰ at 15 °C. It should be noted, however, that the uncertainty in the value at lower temperatures is significant because of the small (<2%) change in Mg concentrations relative to seawater (Fig. 3b and Table 1). Significant deviations from this general trend, toward lower *ε*_solid-fluid_, are observed for extrapolated-SBI fluids from holes 1363F and 1363G, in which temperatures of 7 °C and 6 °C were measured, respectively (Fig. 3a and Table 1)^39^, and for the basement fluids from hole 1024 (23 °C; Fig. 3a and Table 1). The Mg concentration in these extrapolated-SBI fluids also deviates from the general correlation with SBI temperature (Fig. 2b and Table 1), suggesting complex fluid pathways that may have allowed seawater to interact with a slightly warmer crust (ca. 35 °C instead of 6–7 °C; triangles in Fig. 3b) before cooling along the flow path. Such isochemical cooling of the fluid is common in hydrothermal systems^e.g., 45^. On the other hand, the fluids from 1024 (indicated by 23 °C in Fig. 3a and by gray circles in Fig. 3b) may represent mixing between seawater and a warmer hydrothermal endmember (between 40 °C and 60 °C), or a different Mg-removal reaction (with lower fractionation) at this site. Thus, we exclude the samples from 1024 and used 35 °C for 1363F and 1363G in the temperature-dependence function that is presented in Fig. 3b. This temperature-dependence function of *ε*_solid-fluid_ can be described by the uncertainty-weighted linear regression (dotted line in Fig. 3b), as shown by Eq. (1).

1 $$\varepsilon _{{\mathrm{solid}} - {\mathrm{fluid}}} = 2.71 \cdot \frac{1}{{T_{\left( {\mathrm{k}} \right)}}} \cdot 10^3 - 7.81$$

where *T*_(K)_ is the SBI temperature in Kelvin. The suggested *ε*_solid-fluid_ for cold temperatures of 15–25 °C (1.3‰–1.6‰) is in accordance with the maximum enrichment factor previously suggested for authigenic clay formation within marine sediments (1.25‰–1.34‰)^20,46^.

### The Mg flux into the low-temperature hydrothermal sink

The LTH Mg flux into the oceanic crust, *F*_LTH_, can be obtained from the seawater flux that circulates through the oceanic crust on ridge flanks, *F*_sw-LTH_, and the difference in Mg concentration between seawater and the discharging LTH fluids ([Mg]_sw_ − [Mg]_LTH_). This difference can be also defined as (1 − *f*_Mg_)∙[Mg]_sw_, where *f*_Mg_ is the remaining fraction of Mg in the returning fluid relative to seawater, to give:

2 $$F_{{\mathrm{LTH}}} = F_{{\mathrm{sw}} - {\mathrm{LTH}}} \cdot \left( {1 - f_{{\mathrm{Mg}}}} \right) \cdot \left[ {Mg} \right]_{{\mathrm{sw}}}$$

**Fig. 4:**
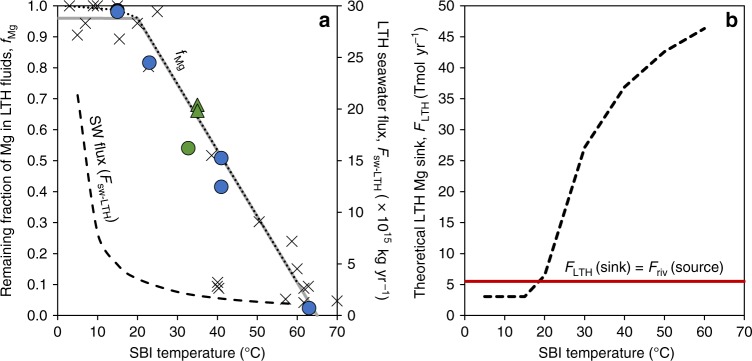
Mg removal by the low-temperature hydrothermal (LTH) system. A theoretical Mg-removal flux is calculated from Eq. (2) assuming different theoretical globally averaged sediment–basement interface (SBI) temperatures. **a** The flux of circulating seawater through the LTH system, (*F*_sw-LTH_; black dashed line) and the remaining fraction of Mg in the LTH fluids (*f*_Mg_; black-dotted line) were used to calculate the Mg flux, *F*_LTH_, in panel b. *F*_sw-LTH_ was calculated using a global heat loss of 8.1 TW, a heat capacity of 4J (g∙°C)^−1^ and a bottom seawater temperature of 2 °C. Colored data points are measured *f*_Mg_ data used in current study (Table 1 and Supplementary Table 1): basement fluids (blue), extrapolated-SBI fluids (green), and temperature-corrected (triangles). The pore-water sample from Site 1363G (see Supplementary Table 1) was collected right at the SBI and it is thus shown in green. Other literature data^30,47^ are shown by X signs. Errors are within the symbols. Gray-solid line is a correlation of *f*_Mg_ literature data after Fisher and Wheat^29^, and Higgins and Schrag^40^. **b** A theoretical global Mg-removal flux by LTH systems (*F*_LTH_; black dashed line) as a function of average SBI temperature. The red horizontal line is the upper limit, at which the low-temperature hydrothermal Mg output matches the riverine input (*F*_LTH_ = *F*_riv_ = 5.5 Tmol yr^−1^). Any *F*_LTH_ higher than the red line requires a rapid depletion of oceanic Mg, a scenario that is incompatible with oceanic Mg concentration records^e.g., 49,50^. This in turn implies that the average SBI temperature must be <20 °C.

As has been shown previously, both *F*_sw-LTH_ and [Mg]_LTH_ (and therefore also *f*_Mg_) depend on temperature at the SBI (Fig. 2b)^e.g., 27,29,30,33,34,47^. *F*_sw-LTH_ can be estimated from the global heat loss via ridge-flank hydrothermal systems, which has been estimated to be 8.1 TW^27^, and the temperature difference between bottom seawater (2 °C) and the SBI temperature^e.g., 29,34^. Thus, we calculated a corresponding *F*_sw-LTH_ for each theoretical heat-loss-weighted average SBI temperature between 2 °C and 60 °C (black dashed line in Fig. 4a).

The temperature dependence of *f*_Mg_ in LTH fluids has been described previously^29^ and is shown in Fig. 4a, where *f*_Mg_ decreases dramatically at temperatures above 15–20 °C. We use the linear correlation suggested by Higgins and Schrag^40^ (gray-solid line in Fig. 4a) to describe *f*_Mg_ between 20 °C and 65 °C. By contrast, at cold temperatures (≤15°C–20 °C) the change in *f*_Mg_ is small, close to the analytical detection limit, and more variable (Fig. 4a)^29^. For these cold temperatures, we estimate *f*_Mg_ (black-dotted line in Fig. 4a) using a linear function between seawater and the coldest LTH fluids in this study (Dorado springs; Supplementary Table 1; ~15 °C and the minimum [Mg]_LTH_ measured there: 52.2 mmol kg^−1^)^34^. The resulting *F*_LTH_ between 2 °C and 15 °C (Eq. 2) is ~60% of the riverine flux (black dashed line in Fig. 4b). At SBI temperatures ≥20 °C, this flux increases dramatically, to above 100% of the riverine flux (black dashed line in Fig. 4b). Given that this higher temperature (≥20 °C) flux would lead to rapid depletion of the oceanic Mg reservoir, the heat-loss-weighted average temperature at the SBI must be <20 °C (equivalent to a water flux, *F*_sw-LTH_, >3.6∙10^15^ kg yr^−1^), in accordance with previous suggestions^e.g., 32,33,48^. This further suggests that the Mg concentration of the ocean is very sensitive to the temperature of the LTH system. For example, an SBI temperature of about ~10 °C higher than the modern temperature, as suggested by Gillis and Coogan^48^ for the Cretaceous, would deplete the entire seawater Mg reservoir within a few Myr. Evidence from fluid inclusions in halite rule this out^e.g., 49,50^, requiring an efficient negative feedback mechanism that is still unknown.

### The oceanic Mg budget in the past ~20 Myr

**Fig. 5:**
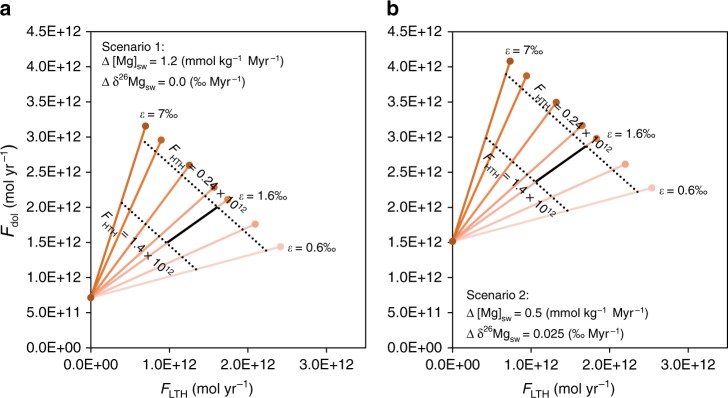
Results of Mg budget calculations. The calculations are done by Eqs. (3) and (4). The solid black line shows the calculated Mg flux into the dolomite sink, *F*_dol_, and the low-temperature hydrothermal (LTH) sink, *F*_LTH_, given the isotope fractionation by the LTH system, *ε*_LTH-sw_, is 1.6‰ and the Mg flux into the high-temperature hydrothermal (HTH) sink, *F*_HTH_, is in the range of 0.24–1.4 Tmol yr^−1^. The orange lines show the results for different *ε*_LTH-sw_ values in the range of 0.6–7‰ (shown: 0.6, 1, 1.6, 2, 3, 5, 7‰). The black-dotted lines define the possible range of *F*_dol_ and *F*_LTH_ given the allowed range in *F*_HTH_. Two extreme scenarios are presented, equivalent to: (**a**) a rapid increase in [Mg]_sw_ (1.2 mmol kg^−1^ in Myr) with no change in seawater δ^26^Mg. This scenario results in minimum output fluxes; and (**b**) a slow rise in [Mg]_sw_ (0.5 mmol kg^−1^ in Myr) and larger changes in seawater δ^26^Mg (0.025 ‰ Myr^−1^). This scenario results in maximum output fluxes.

In this section, we present a sensitivity analysis with a reasonable range of HTH Mg flux, *F*_HTH_ = 0.24–1.4 Tmol yr^−1^ (taken from the literature; Supplementary Table 2), to assess the impact of the LTH Mg concentration and isotope fractionation on global budgets, and by inference the required dolomite output to balance both concentration and isotope composition. Based on the conclusions from the section above, calculations were conducted for an average SBI temperature of ~15 °C, which corresponds to *F*_sw-LTH_ = 4.9∙10^15^ kg yr^−1^. However, due to the high sensitivity of the Mg flux, *F*_LTH_, to the measured Mg concentration of the coldest LTH fluids studied here (Dorado springs; [Mg]_LTH_ = 52.2 mmol kg^−1^)^34^, Mg budget calculations allowed *F*_LTH_ to vary between 0.0% and 100% of the output fluxes, which means that the average [Mg]_LTH_ is between 51.8 and 53 mmol kg^−1^ (within 2% of the seawater value). In addition, given the generally uniform rock-type composition of MORBs and the nature of the LTH circulation, which is confined to the upper few hundred meters of volcanic crust^51^, our isotopic results were assumed to represent the global LTH effect and the LTH isotope fractionation was calculated at ~15 °C using Eq. (1) to be *ε*_LTH-sw_ = 1.6‰. Consistent with the large errors on the measured data, we also present the results for a much wider range of *ε*_LTH-sw_ values (0.6–7‰; Fig. 5).

As noted above, the modern ^26^Mg-enriched ocean (−0.83‰)^18,19^ relative to riverine Mg sources (−1.09‰)^15^ requires a significant sink of Mg that preferentially removes the lighter Mg isotopes (i.e., negative *ε*_sink-sw_) or an additional ^26^Mg-enriched input. As shown in this study, the isotope fractionation during low-temperature interaction with the oceanic crust is in the opposite direction, with a range of positive *ε*_LTH-sw_ depending on the temperature of the LTH system (Fig. 3 and Table 1). This requires an even larger ^26^Mg-depleted sink or ^26^Mg-enriched source than previously thought.

Currently, the only known significant ^26^Mg-depleted sink results from the formation of dolomite^e.g., 15,20,40^. Marine calcite is also a ^26^Mg-depleted sink, but the vast majority of calcite sediments forming today are low in Mg, so that the Mg flux into marine calcite is also considered to be very low (ca. 0.3 Tmol yr^−1^)^e.g., 2–4^. Although some previous estimations of the Mg flux resulting from ion exchange with clays suggest a small Mg output (< 0.3 Tmol yr^−1^)^e.g., 2,15,52^, other studies of soils and sediments suggest that such reactions with clays may supply ^26^Mg-enriched Mg to the fluid phase^42,53,54^. Thus, more experimental and field research in the marine realm is needed to understand the isotopic effects of such processes.

To calculate the dolomite Mg-removal flux required to balance the Mg isotope budget of the ocean, we apply two mass-balance equations describing the Mg elemental and isotopic budgets of the ocean:

3 $$M_{{\mathrm{sw}}} \cdot \frac{{d[Mg]_{{\mathrm{sw}}}}}{{dt}} = F_{{\mathrm{riv}}} - F_{{\mathrm{dol}}} - F_{{\mathrm{HTH}}} - F_{{\mathrm{LTH}}}$$

and

4 $$N_{{\mathrm{Mg}}} \cdot \frac{{d\delta _{{\mathrm{sw}}}}}{{dt}} = F_{{\mathrm{riv}}} \cdot \left( {\delta _{{\mathrm{riv}}} - \delta _{{\mathrm{sw}}}} \right) - F_{{\mathrm{dol}}} \cdot \varepsilon _{{\mathrm{dol}} - {\mathrm{sw}}} - F_{{\mathrm{LTH}}} \cdot \varepsilon _{{\mathrm{LTH}} - {\mathrm{sw}}}$$

Where *M*_sw_ is the mass of water in the ocean, [Mg]_sw_ is the Mg concentration in seawater, *t* is time, and *F*_riv_, *F*_dol_, *F*_HTH_, and *F*_LTH_ are the oceanic Mg fluxes (mol yr^−1^): riverine input, dolomite formation, HTH, and LTH systems, respectively. *N*_Mg_ is the Mg inventory of seawater. *δ*_sw_ and *δ*_riv_ are δ^26^Mg values of seawater and rivers, respectively, and *ε*_dol-sw_ and *ε*_LTH-sw_ are the isotope enrichment factors during dolomite formation and LTH alterations, respectively. The HTH alterations remove Mg quantitatively and thus have no effect on the isotope budget (*ε*_HTH-sw_ = 0).

Equations (3) and (4), coupled with parameters listed in the Supplementary Information (Supplementary Table 2), allow us to assess the dolomite (*F*_dol_) and LTH (*F*_LTH_) flux. Parameters in Supplementary Table 2 were chosen to describe the average budget in the Neogene, ca. 20 Myr (roughly 1.5 times the residence time of Mg in the ocean, ~13 Myr)^e.g., 16^. The sedimentary authigenic clay sink is at least an order of magnitude smaller (0.2 Tmol yr^−1^)^46^ than the LTH flux calculated below, whereas the isotope fractionation (+1.3‰)^20,46^ is similar. Thus here, Mg removal into authigenic clays is not distinguished from LTH removal. The above constrained seawater flux through LTH systems, *F*_sw-LTH_, and isotope fractionation, *ε*_LTH-sw_, set limits on the current rate of dolomite formation that is required to balance oceanic Mg concentration and isotope budgets.

For any given *ε*_LTH-sw_, the *F*_dol_ required by Eq. (4) is a linear function of *F*_LTH_. For higher *ε*_LTH-sw_, a given *F*_LTH_ requires higher dolomite fluxes (orange lines in Fig. 5). Also, for a given *F*_HTH_, the *F*_dol_ required by Eq. (3) is a linear function of *F*_LTH_, with a constant slope of −1 and a decreasing required *F*_LTH_ as *F*_HTH_ increases (black-dotted lines in Fig. 5). Hence, for each *ε*_LTH-sw_ and *F*_HTH_, these two equations provide unique *F*_dol_ and *F*_LTH_. As the records of the past ~20 Myr involve some uncertainty in the evolution of [Mg]_sw_ and δ^26^Mg (defined in Supplementary Table 2), we conducted calculations for two extreme scenarios as follows: (1) one with a minimum output flux (Fig. 5a), in which the rise in [Mg]_sw_ is at a maximum, at 1.2 mmol (kg∙Myr)^−1^, and the change in seawater δ^26^Mg value is at a minimum, at 0 ‰ Myr^−1^; and (2) a maximum output flux scenario (Fig. 5b), in which the rise in [Mg]_sw_ is at a minimum, at 0.5 mmol (kg∙Myr)^−1^, and the change in seawater δ^26^Mg value is at a maximum, at 0.025 ‰ Myr^−1^.

The possible ranges for the oceanic Mg fluxes are summarized in Table 2. The resulting *F*_LTH_ (1.0–1.7 Tmol yr^−1^; ~20–40% of the total Mg outputs) imply that the average [Mg]_LTH_ is between 52.5 and 52.7 mmol kg^−1^, which is analytically indistinguishable from Mg concentration in seawater ([Mg]_sw_ = 52.9 mmol kg^−1^), stressing the difficulty in identification and measurement of Mg removal by these LTH fluids^34^. This range of *F*_LTH_ is in line with several recent studies of the oceanic Mg isotope budget^e.g., 40^, but is significantly higher than those suggested by earlier studies (0.4 Tmol yr^−1^)^e.g., 2^ and by other recent studies (0.67 Tmol yr^−1^)^e.g., 43^.

**Table 2:** Oceanic magnesium fluxes (Tmol yr^−1^).

	This study Minimum fluxes scenario	This study Maximum fluxes scenario	This study 50% Silicate weathering scenario	Wilkinson and Algeo^25^: Modern (average Phanerozoic)	Holland^2^	MAGic: Arvidson et al.^3,5^: Quaternary average	Higgins and Schrag^40^: Cenozoic range	Gothmann et al.^41^: Cenozoic range
Input rivers	5.5	5.5	3.9	5.2 (5.4)	6.1	4.9	4.0–4.7	4.5
Increment to seawater	1.6	0.7	0.7–1.6	0	1.7	0	0.4–1.4	0.4–1.4
Total outputs	3.9	4.8	2.3–3.2	5.2	4.4	4.9	–	–
High-T hydrothermal	0.24–1.4	0.24–1.4	0.24–1.2^a^	5.1 (2.2)^b^	2.0	2.6^b^	1.4–1.6	1.7–2.5^b,c^
Low-T hydrothermal	1.0–1.6^c^	1.1–1.7^c^	0.0–0.6^c^	–	0.4^c^	–	0.6–1.5^c^	–
Dolomite	1.5–2.0	2.3–2.9	1.1–2.4	0.1 (1.8)	1.7	0.1	0.75–1.0	0.75–1.75
Other	–	–	–	–	0.3 (Cation exchange)	2.2 (Chlorite)	–	–

^a^Limited by the lower input flux

^b^Includes also the low-temperature hydrothermal Mg sink

^c^Includes also authigenic clays formation and reverse weathering

The resulting dolomite flux (*F*_dol_ = 1.5–2.9 Tmol yr^−1^; ~40–60% of the total Mg outputs) is higher than those previously suggested (Table 2). The results of this study show that it is impossible to keep the δ^26^Mg value of the ocean close-to-constant without a significant rate of dolomite formation and an even larger rate is required to keep the oceanic δ^26^Mg value (−0.83‰)^18,19^ higher than the riverine inputs (−1.09‰)^15^. For the very low dolomite formation flux of the conventional paradigm^e.g., 3,5,25^, the δ^26^Mg value of the ocean would decrease dramatically (decrease of >1‰ within ~20 Myr). Instead, available records of the last ~20 Myr show that seawater δ^26^Mg is close-to-constant or even slightly increasing^40,41^. This requires a dolomite flux similar to or even higher than the Phanerozoic average (1.8 Tmol yr^−1^; based on mass-age distribution of carbonate rocks^25^).

One additional uncertainty in these calculations is the size and isotope composition of the riverine flux of Mg, here assumed to be the same as modern for the past 20 Myr. There has been recent debate over whether there have been secular changes in the rate and style of chemical weathering, particularly silicate weathering, both on glacial-interglacial timescales in the Quaternary^e.g., 17,55^ and over the Cenozoic^e.g., 56^. As an example, a reduction in silicate weathering by 50% would require lower calculated *F*_LTH_ (0.0–0.6 Tmol yr^−1^), but similar *F*_dol_ (1.1–2.4 Tmol yr^−1^; Table 2). This is because lower Mg input from silicate rocks would lower *δ*_riv_ (Supplementary Table 2), which needs to be compensated by a dolomite formation rate that is high enough—really to keep the oceanic δ^26^Mg value close-to-constant.

## Discussion

Despite more than a century of research, the origin of dolomite remains the subject of considerable debate, a debate often referred to as the “Dolomite Problem”^e.g., 5,10,57–59^. Laboratory experiments indicate that dolomite formation at Earth surface conditions is kinetically inhibited^e.g., 58^. However, several studies have shown that non-ordered dolomite can potentially precipitate from seawater as a result of geomicrobiological processes^59^ and it seems unlikely that all ancient dolomite formed through high-temperature metamorphic reactions^e.g., 57,60^. Compilations of carbonate rock records have suggested that despite its abundance in most of the Phanerozoic Eon, dolomite is a scarce sediment during the Cenozoic Era^e.g., 23^. Modern dolomite formation is considered to be very rare (<2% of the oceanic Mg output fluxes) and mainly restricted to shallow-water evaporitic environments^e.g., 3,25^. These observations have led to the commonly held view that dolomite formed abundantly in the past, during periods characterized by high sea levels and spatially extensive shallow-evaporitic environments^e.g., 23^. Thus, dolomite is also commonly related to periods of higher atmospheric CO_2_ and higher temperatures, with lower formation rates in colder periods with lower sea level, such as the Cenozoic Era^e.g., 10,57^. Consequently, some authors ascribe the increase in seawater Mg concentration over the Cenozoic to the lower dolomite content of more recent carbonate sediments^e.g., 23^, associated with an increase in the deposition of carbonates in deep-sea sediments^24^, and the difficulty of dolomitizing deep-sea CaCO_3_ by reaction with cold, unevaporated seawater^e.g., 2,3,5,10^.

However, the Mg isotope fractionation data obtained here, combined with the results of other recent Mg isotopes studies^e.g., 15,20,40–42^, do not support this view. Instead, it is suggested here that the average Mg-removal flux to dolomite during the past ~20 Myr is similar or higher than the Phanerozoic average^25^. This implies that our traditional view of the conditions under which dolomite forms needs to be revised and points to the existence of a hidden pool of modern dolomite. Assuming that most modern shallow-water sedimentary systems have been extensively explored, the hidden pool may reside in the deep ocean. The presence of dolomite (layers and/or disseminated dolomite) has been reported in several cores collected in the framework of ocean drilling projects^e.g., 20,42,61,62^ and it is possible that more dolomite than previously thought exists in this relatively unexplored deep-marine environment. Moreover, it has recently been demonstrated that high temperatures and evaporated high Mg/Ca seawater are not a key requirement for dolomite formation, and that diagenetic replacement of aragonite by dolomite can occur at temperatures of about 4 °C^63^. Dolomitization of Ca-carbonates could, therefore, take place in the cold pore waters of deep-ocean sediments. Alternatively, as recently proposed by Ryb and Eiler^64^, massive marine dolomites may form in carbonate platforms, at great depth and at elevated burial temperatures over timescales of 100 Myr. If the source of the Mg is Cenozoic seawater and if the Mg-stripped water is returned to the ocean, for example by “Kohout” convection^e.g., 65^, this proposal could also reconcile the apparent conflict between the age distribution of dolomite and the oceanic Mg isotope record. Regardless of whether the hidden formation site of dolomite is located, or whether the dolomite is primary, early diagenetic, or a late-stage replacement of pre-existing Ca-carbonates, Mg isotopes indicate that a significant amount of dolomite formed during the past ~20 Myr, with Mg deriving from seawater.

We have determined, for the first time, the Mg isotope fractionation associated with the important but enigmatic oceanic Mg sink at LTH systems, by measuring the Mg isotope composition (δ^26^Mg) of LTH fluids. The results indicate a temperature-dependent preferential removal of heavy isotopes into the oceanic crust, enriching the ocean with light isotopes. In addition, we show that the global average temperature of the LTH system must be <20 °C to meet oceanic Mg balance requirements. Based on the above findings, we suggest that the global Mg isotope fractionation associated with the LTH sink, *ε*_LTH-sw_, is ~1.6‰. Utilizing this fractionation to constrain the oceanic Mg and Mg isotope budget over the past ~20 Myr requires that a significant amount of dolomite (1.5–2.9 Tmol yr^−1^; 40–60% of the total oceanic Mg output flux) has formed since the beginning of the Neogene, a period characterized by lower seawater level and relatively cold climatic conditions. Thus, the commonly held view that modern dolomite is rare, and that dolomite abundance in ancient rocks corresponds to periods of higher atmospheric CO_2_—higher sea levels—higher temperatures need to be revised. The depositional settings of such a large volume of recent dolomite remains unknown, but we suggest that this hidden modern dolomite may reside in the sediments of the deep ocean.

## Methods

### Materials

Basement (formation) fluids were extracted from IODP Holes 1362A and 1362B, which were drilled in 2010 during IODP expedition 327 on the eastern flank of Juan de Fuca ridge^38^. The boreholes were sealed at the seafloor and throughout the sediment section but remained open to basaltic basement. In each Hole, a Circulation Obviation Retrofit Kit, CORK, was installed. Fluids were sampled using the ROV *Jason II* during Atlantis Expedition AT 18-07^38^. The overpressured basement fluids were sampled directly from the well head valve, thus eliminating the potential for mixing with bottom seawater.

Basement fluids were also extracted from ODP Sites 1024 and 1025, which were drilled in 1996 during ODP Leg 168^e.g., 36,37^, on the eastern flank of Juan de Fuca ridge. The boreholes were instrumented with a CORK and sealed at the seafloor and throughout the sediment section but remained open to basaltic basement. These CORKs also included modular fluid samplers driven by osmotic pumps (OsmoSamplers)^e.g., 36,37^. Samples from Site 1024 used in this study were recovered from the OsmoSampler in 1999. The OsmoSampler from Site 1025 was not recovered because poor hole conditions entombed the samplers. However, the overpressured basement fluids in Site 1025 were sampled in 1999 and 2000: in 1999, three fluid samples were collected from the fluid sample port at the well head after allowing the fluids to vent for 3 h. In 2000, about twelve borehole volumes were allowed to vent before four samples were collected. Samples from ODP Site 1025, were partially diluted with bottom seawater because the manifold was not sealed perfectly^37^. Thus, following Wheat et al.^37^, the lowest Mg concentration measured at each of the two dives (22.0 and 26.9 mmol kg^−1^; Supplementary Table 1) is used as the basement fluid endmember to account for the dilution of borehole samples with bottom seawater while sampling.

Basement fluids were also sampled from cool seafloor hydrothermal springs from Dorado outcrop, Cocos Plate, eastern flank of the East Pacific Rise, during expeditions AT26-09 and AT26-24^34^. Discrete measurements of temperature and dissolved oxygen were used to locate the hydrothermal springs. Only after confirming the warmest temperatures or low dissolved oxygen concentrations were discrete fluid samples collected. Discrete fluid samples were collected by placing a sampler inlet directly in shimmering spring discharge or within the opening of an inverted funnel, which was used to minimize seawater entrainment during sampling^34^.

**Fig. 6:**
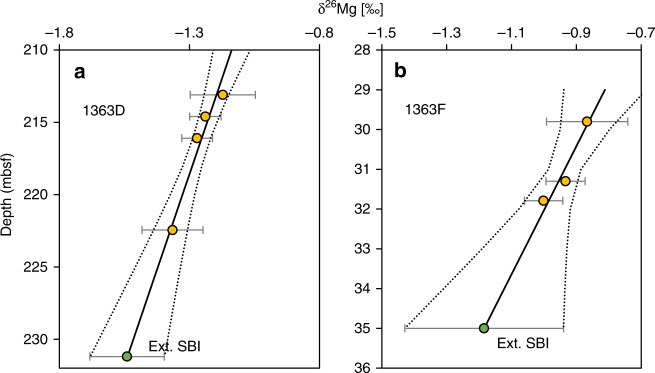
Extrapolation of pore-water data. The δ^26^Mg values in pore water are linearly extrapolated to the sediment-basement interface (SBI) depth. **a** Hole U1363D and **b** Hole U1363F. Pore-water data are shown in yellow. Extrapolated-SBI data are shown in green. Error bars are 2 SD.

Pore water from the sediments recovered at IODP Sites U1363A-F were extracted by squeezing selected parts of the sediment cores during IODP Expedition 327 in 2010^e.g., 39^. The IODP Site U1363 boreholes are located on a seismic transect (GeoB00-170) close to the Grizzly Bare outcrop, a site where seawater recharges the basement^39,66,67^. Pore waters may be affected by sediment diagenetic processes and diffusive fluxes from the overlying seawater and underlying basaltic basement fluids. Therefore, following Wheat et al.^39^ and others, the isotope composition of upper-basement fluid in Holes U1363D and U1363F was linearly extrapolated from the deepest samples, based on the known depth of the SBI (Fig. 6). δ^26^Mg values of pore water from Hole U1363B show small variations and opposite correlation with depth. Therefore, an extrapolated value was not calculated. In the case of Hole U1363G, the deepest sample was collected at the SBI, so the composition of this fluid is considered to be that of the upper-basement fluid. Wheat et al.^37^ validated this approach for concentrations by comparing the concentrations of various elements in basement fluid from Hole 1025 with the concentration calculated by extrapolating pore-water compositions to the SBI. They found that for most elements (including Mg) the difference between these two methods was <5%.

### Analytical methods

Samples were analyzed for δ^26^Mg and δ^25^Mg values at ETH, Zurich. An aliquot of each sample was dried down and re-dissolved in 0.5 M HCl. 200 µl containing ~1 µg Mg was then loaded onto 30 ml Savillex Microcolumns (6.4 mm ID × 9.6 mm OD) with ~1 ml (3 cm high) Bio-Rad AG® 50W-X12 (200–400 mesh) resin for Mg purification. The matrix was rinsed with 45 ml 0.5 M HCl and Mg was collected by 5 ml 0.5 M HCl and 6 ml 2.0 M HCl. The total procedural blank for Mg isotope ratio measurements by this method is <5 ng Mg, which is insignificant compared to the amount of Mg loaded onto columns (<0.5%). Splits of the elution were collected before and after the Mg collection, to ensure that close to 100% Mg yield was achieved, i.e., <0.3% of the processed Mg was detected in these splits. All concentrations were determined by a Thermo Scientific Element XR ICP-MS.

Magnesium isotope ratios were measured using a Thermo Scientific Neptune MC-ICP-MS with an “H” Ni skimmer cone and conventional Ni sample cone interface. A purified sample solution of ~200 p.p.b. Mg in 2% v/v HNO_3_ was introduced via an Elemental Scientific (ESI) Apex-Q desolvating system coupled with a PFA nebulizer with a nominal uptake rate of 50 ml min^-1^. Mg isotope ratios were measured in low mass resolution mode, with all intensities at m/z 24, 25, and 26 measured simultaneously in separate Faraday cups (H3, Center, L3). These conditions gave typical signals of 15–20 V on m/z 24. The on-peak background in 2% v/v HNO_3_ was repeatedly recorded during the sequence and was ≤0.2% of the samples intensities at m/z 24, 25, and 26, and therefore considered as insignificant. Each individual measurement consisted of 25 ratios, each of 4 s integration time (a total time of 100 s). Each sample was measured by several (*n*) standards-sample brackets, and the δ^26^Mg and δ^25^Mg values are reported relative to the delta-zero standard, DSM3:

5 $${\delta}^{x}Mg = {\delta}^{x/24}Mg_{{\mathrm{sample}}/{\mathrm{DSM}}3}=\left[\frac{\left({\,\!}^{x}Mg/{\,\!}^{24}Mg\right)_{\mathrm{sample}}}{\left({\,\!}^{x}Mg/{\,\!}^{24}Mg\right)_{\mathrm{DSM3}}} -1\right]$$

where *x* denotes either 26 or 25 and DSM3 is the mean value of the two bracketing standards measured before and after the sample, respectively. Multiplication of equation (5) with a factor 1000 gives the per mil (‰) deviation relative to DSM3.

Unprocessed Cambridge-1 (pure Mg solution) was measured during each measurements session and gave results identical to the values reported in the literature (Supplementary Table 3)^e.g., 68^. To validate the whole procedure, including column chemistry and MC-ICPMS measurements, seawater was processed and measured together with each batch of samples and the pure Mg solutions, DSM3 and Cambridge-1, were processed through columns and measured. In addition, three matrix reference materials, JDo-1, CRM-512 and DSW-1, were analyzed. All these gave results identical to the previously reported values, within errors (Supplementary Table 3)^e.g., 19,69^. Furthermore, the δ^25^Mg versus δ^26^Mg results determined in this study plot on a single line with a slope of 0.519 (Supplementary Fig. 1), suggesting no major influence of isobaric interferences on the measured Mg isotope ratios.

### Estimations of isotope fractionation

To define the isotope fractionation that accompanies Mg removal from seawater, we use the enrichment factor, *ε*_solid-fluid_ = (*α*_solid-fluid_ − 1)∙1000, where *α*_solid-fluid_ = (^26^Mg/^24^Mg)_solid_/(^26^Mg/^24^Mg)_fluid_. The *ε*_solid-fluid_ was calculated for each sample assuming a Rayleigh distillation process:

6 $$\frac{{\delta ^{26}Mg_{{\mathrm{sample}}} + 1000}}{{\delta ^{26}Mg_{{\mathrm{SW}}} + 1000}} = f_{{\mathrm{Mg}}}^{\left( {\varepsilon /1000} \right)}$$

where *f*_Mg_ is the remaining fraction of Mg (i.e., Mg concentration in the sample divided by Mg concentration in bottom seawater), δ^26^Mg_SW_ equals −0.83‰ and δ^26^Mg_sample_ is the measured isotopic composition of each sample.

## Supplementary information

Supplementary Information

## Data Availability

The authors declare that the data supporting the findings of this study are available within the paper and its Supplementary Information files.
