# Supplementary material for: New isotope constraints on the Mg oceanic budget point to cryptic modern dolomite formation
Source: Nat Commun. 2019 Dec 11;10:5646. doi: 10.1038/s41467-019-13514-6 (PMC6906300; doi:10.1038/s41467-019-13514-6)
Supplement: Supplementary file 1 — Supplementary Information [file 41467_2019_13514_MOESM1_ESM.pdf]

New isotope constraints on the Mg oceanic budget point to cryptic modern dolomite formation

Shalev et al.

Supplementary Information

Supplementary Figures

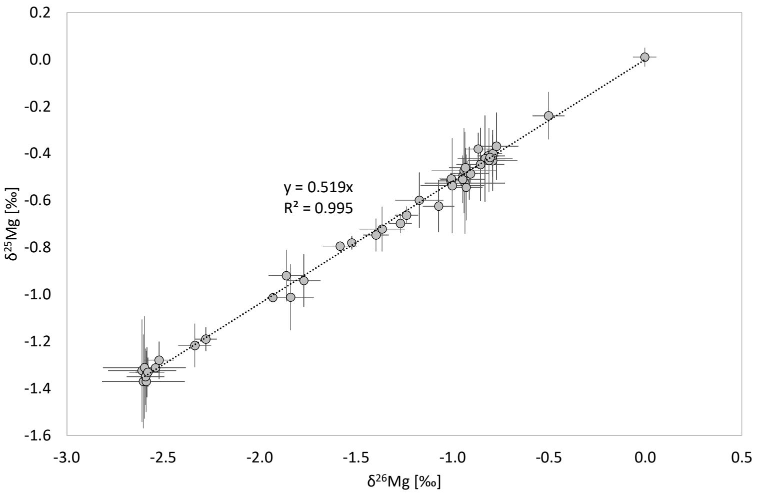

Supplementary Figure 1 δ^{25}Mg versus δ^{26}Mg values determined in this study. Error bars are 2SD of each result. The data are in accordance with a regression line (dotted) that crosses the origin and has a slope of 0.519, typical of mass-dependent fractionation of terrestrial material.

Supplementary Tables

Supplementary Table 1: Sampling locations and description of the studied LTH fluids.

Sample	Sampling year	Expedition	Hole	Latitude (°N)	Longitude (°W)	Plate	Sediment cover (m)	Depth of sampling (mbsf)	Temp. at SBI (°C)	Mg	Ca	Na	K
										(mmol kg^{-1})			
Basement (formation) fluids													
1362A-MVBS-13*	2014	IODP 327	1362A	47° 45.663'	127° 45.672'	Juan de Fuca	236	528	64	1.3	57.6	467	6.9
1362B-sled #5*	2011	IODP 327	1362B	47° 45.500'	127° 45.731'	Juan de Fuca	242	359	64	1.3	56.8	462	6.8
1024-46†	1999	ODP Leg 168	1024	47° 54.531'	128° 45.005'	Juan de Fuca	152	170	23	43.2	14.1	ND§	10.5
1024-90†	1999	ODP Leg 168	1024	47° 54.531'	128° 45.005'	Juan de Fuca	152	170	23	43.2	14.1	ND§	ND§
1025-3472-#3-acidified#	1999	ODP Leg 168	1025	47° 53.25'	128° 38.88'	Juan de Fuca	101	147	40.5	22.0	25.8	341	ND§
1025-3472-#2-unacidified#	1999	ODP Leg 168	1025	47° 53.25'	128° 38.88'	Juan de Fuca	101	147	40.5	43.4	18.4	427	ND§
1025-3608-blue#	2000	ODP Leg 168	1025	47° 53.25'	128° 38.88'	Juan de Fuca	101	147	40.5	26.9	32.6	437	ND§
1025-3608-red#	2000	ODP Leg 168	1025	47° 53.25'	128° 38.88'	Juan de Fuca	101	147	40.5	27.7	32.8	446	ND§
Springs fluids**													
4775-6-Dorado	2014	AT26-24	NA§	09° 04.949'	087° 05.727'	Cocos (eastern flank of the East Pacific Rise)	0	0	15	52.5	10.2	466	10.1
4777-7-Dorado	2014	AT26-24	NA§	09° 04.948'	087° 05.728'	Cocos (eastern flank of the East Pacific Rise)	0	0	15	52.4	10.2	472	10.2
4775-9-Dorado	2014	AT26-24	NA§	09° 04.949'	087° 05.727'	Cocos (eastern flank of the East Pacific Rise)	0	0	15	52.3	10.2	464	10.2
4777-6-Dorado	2014	AT26-24	NA§	09° 04.948'	087° 05.728'	Cocos (eastern flank of the East Pacific Rise)	0	0	15	53.3	10.2	464	10.1
Pore Water††													
1363G-3H1	2010	IODP 327	1363G	47° 17.312'	128° 02.170'	Juan de Fuca	17.5	17.5	6.1	36	21	473	9.2
1363D-4X1	2010	IODP 327	1363D	47° 17.572'	128° 01.760'	Juan de Fuca	231	213	32.7	33.3	33.4	473	5.8
1363D-4X2	2010	IODP 327	1363D	47° 17.572'	128° 01.760'	Juan de Fuca	231	215	32.7	33.3	34.7	480	6.0
1363D-4X3	2010	IODP 327	1363D	47° 17.572'	128° 01.760'	Juan de Fuca	231	216	32.7	34.7	36.1	486	5.5
1363D-5X1	2010	IODP 327	1363D	47° 17.572'	128° 01.760'	Juan de Fuca	231	222	32.7	31.3	35.8	478	6.1
**1363D**			**Extrapolated SBI**				**231**	**231**	**32.7**	**28.6**	**35**	**471**	**4.6**

Sample	Sampling year	Expedition	Hole	Latitude (°N)	Longitude (°W)	Plate	Sediment cover (m)	Depth of sampling (mbsf)	Temp. at SBI (°C)	Mg	Ca	Na	K
										(mmol kg^{-1})			
1363B-4H5	2010	IODP 327	1363B	47° 17.352'	128° 02.106'	Juan de Fuca	57	29	nd	50.6	10.0	497	11.7
1363B-7X1	2010	IODP 327	1363B	47° 17.352'	128° 02.106'	Juan de Fuca	57	44	nd	39.5	20.3	494	11.2
1363B-8X2	2010	IODP 327	1363B	47° 17.352'	128° 02.106'	Juan de Fuca	57	50	12.2	36.8	20.7	492	11.1
1363B-8X3	2010	IODP 327	1363B	47° 17.352'	128° 02.106'	Juan de Fuca	57	52	12.2	38.2	21.9	490	10.8
**1363B**			**Extrapolated SBI**				**57**	**57**	**12.2**	**35**	**23.3**	**474**	**8.5**
1363F 4H1	2010	IODP 327	1363F	47° 17.326'	128° 02.137'	Juan de Fuca	35	30	7.2	37.0	19.7	477	10.4
1363F 4H2	2010	IODP 327	1363F	47° 17.326'	128° 02.137'	Juan de Fuca	35	31	7.2	37.8	20.8	498	11.0
1363F 4H3 IW29	2010	IODP 327	1363F	47° 17.326'	128° 02.137'	Juan de Fuca	35	32	7.2	36.7	21.1	499	10.8
**1363F**			**Extrapolated SBI**				**35**	**35**	**7.1**	**35**	**22.6**	**464**	**8.9**

[*] Data from: Fisher et al., 2012; Wheat et al., 2003; Wheat et al., 2004[1–3]; Chemical data from current study, Wheat et al., 2017; Wheat et al., 2013[4,5].

[†] Data from Wheat et al., 2003[2].

[§] ND is not determined; NA is not applicable.

[#] Data from Wheat et al., 2004[3]; Chemical data from current study.

[**] Data from Wheat et al., 2017[4].

[††] General data and extrapolated values from Wheat et al., 2013[5]; Chemical data from IODP Website.

Supplementary Table 2. Parameters used in the calculations of the oceanic magnesium budget

Parameter	Description	Value/ Range	Units	Comments and References
Mg budget				
M_{sw}	Water mass of the ocean	$1.37 \cdot 10^{21}$	kg	Modern
$\frac{d[Mg]_{sw}}{dt}$	Change in seawater Mg concentration during a period of time (*dt*)	0.5 – 1.2	mmol $(kg \cdot Myr)^{-1}$	Approximation after Zimmermann, 2000; Lowenstein et al., 2001; Horita et al., 2002; Brennan et al., 2013[6–9]
F_{riv}	Mg input riverine flux (Glacial-interglacial cycle)	5.5 (3.9)	Tmol yr^{-1}	Modern: Berner and Berner, 1996; Elderfield and Schultz, 1996; Arvidson et al., 2006[10–12]; Glacial-interglacial cycle: calculated taking into account 50% of the silicate weathering flux (after Vance et al., 2009[13]) and δ^{26}Mg values of silicate and carbonate rocks are -0.3‰ and -2.25‰, respectively (after Higgins and Schrag, 2015[14])
$F_{sw\text{-}HTH}$	Seawater flux through the high-temperature (axial) hydrothermal system	$0.5 \cdot 10^{13}$ – $3 \cdot 10^{13}$	kg(sw) yr^{-1}	e.g., Elderfield and Schultz, 1996; Davis et al., 2003; Mottl, 2003; Teagle et al., 2003; Holland, 2005; compiled in Vance et al. (2009)[11,13,15–18]
F_{HTH}	Mg flux into the oceanic crust at high temperatures	0.24 – 1.4	Tmol yr^{-1}	Based on H_2O flux and 20Myrs average Mg concentration (47 mmol kg^{-1})
$F_{sw\text{-}LTH}$	Seawater flux through the low-temperature (flanks) hydrothermal system	$4.9 \cdot 10^{15}$	kg(sw) yr^{-1}	Calculated from heat flux (after: Fisher and Wheat, 2010; Wheat et al., 2017[4,19]) at average SBI temperature of ~15°C.
F_{LTH}	Mg flux into the oceanic crust at low temperatures	output	Tmol yr^{-1}	Determined in this study.
F_{dol}	Mg flux into dolomite	output	Tmol yr^{-1}	Determined in this study.
Isotopes budget				
N_{Mg}	Mg inventory in sw	$6.4 \cdot 10^{19}$	mol	Calculated from Mg concentration of 47 mmol kg^{-1} (20Myr average).
$\frac{d\delta_{sw}}{dt}$	Change in seawater δ^{26}Mg value, δ_{sw}, during a period of time (*dt*)	0.0 – 0.025	‰ Myr^{-1}	Higgins and Schrag, 2015; Gothmann et al., 2017[14,20] (0-0.5 ‰ $20Myr^{-1}$)
δ_{sw}	Seawater δ^{26}Mg value	-0.83	‰	Foster et al., 2010; Ling et al., 2011[21,22]
δ_{riv}	Weighted average δ^{26}Mg value of rivers (Glacial-interglacial cycle)	-1.09 (-1.41)	‰	Modern: Tipper et al., 2006[23]; Glacial-interglacial cycle: calculated taking into account 50% of the silicate weathering flux (after Vance et al., 2009[13]) and δ^{26}Mg values of silicate and carbonate rocks are -0.3‰ and -2.25‰, respectively (after Higgins and Schrag, 2015[14])
$\varepsilon_{dol\text{-}sw}$	Enrichment factor during dolomite formation	-2.0	‰	Higgins and Schrag, 2010[24]
$\varepsilon_{LTH\text{-}sw}$	Enrichment factor during low-temperature hydrothermal alterations of the oceanic crust	1.6 (0.6 – 7)	‰	This study; At average SBI temperature of ca. 15°C. A wider range is also presented in parentheses.

Supplementary Table 3: $\delta^{26}Mg$ and $\delta^{25}Mg$ results of reference materials.

Material and replicates*	$\delta^{26}Mg$ (‰)	2SD (‰)	$\delta^{25}Mg$ (‰)	2SD (‰)	n
DSM3					
Passed through column	0.00	0.06	0.01	0.04	4
Cambridge-1 (pure Mg)					
A	-2.60	0.21	-1.37	0.20	3
B	-2.59	0.08	-1.37	0.13	4
C	-2.52	0.08	-1.28	0.08	3
D	-2.54	0.05	-1.31	0.02	4
E	-2.61	0.18	-1.32	0.22	4
F	-2.58	0.09	-1.33	0.11	4
G	-2.60	0.21	-1.31	0.22	4
Passed through column	-2.59	0.10	-1.35	0.12	4
**Average**	**-2.58**	**0.06**	**-1.33**	**0.06**	**8**
**Literature†**	**-2.61**	**0.05**	**-1.34**	**0.04**	
Seawater					
A	-0.82	0.06	-0.41	0.03	4
B	-0.79	0.13	-0.43	0.13	4
C	-0.79	0.06	-0.41	0.09	4
D	-0.79	0.05	-0.40	0.05	4
E	-0.83	0.14	-0.42	0.18	4
F	-0.81	0.08	-0.43	0.14	8
**Average**	**-0.81**	**0.03**	**-0.42**	**0.02**	**6**
**Literature§**	**-0.83**	**0.09**	**-0.43**	**0.06**	**90**
Jdo-1 Dolomite					
A	-2.28	0.06	-1.19	0.05	4
B	-2.34	0.09	-1.22	0.09	4
**Literature#**	**-2.35**	**0.15**	**-1.23**	**0.09**	**11**
CRM-512 dolomite					
A	-1.86	0.09	-0.92	0.11	4
B	-1.93	0.02	-1.01	0.02	4
**Literature#**	**-2.03**	**0.17**	**-1.05**	**0.09**	**6**
DSW-1 Dead Sea brine					
A	-0.50	0.08	-0.24	0.10	4
**Literature#**	**-0.58**	**0.12**	**-0.30**	**0.07**	**8**

* Different column chemistry replicates are indicated by A-F, except for replicates A-G of the pure Mg Cambridge-1, which include the MC-ICP-MS measurements only.
† An and Huang (2014), Shalev et al. (2018)[25,26].
§ Ling et al. (2011)[22].
# Shalev et al. (2018)[26].

Supplementary References

1. Fisher, A. T. *et al.* IODP expedition 327 and Atlantis expedition at 18-07: Observatories and experiments on the eastern Flank of the Juan de Fuca Ridge. *Sci. Drill.* 4–11 (2012). doi:10.2204/iodp.sd.13.01.2011

2. Wheat, C. G., Jannasch, H. W., Kastner, M., Plant, J. N. & DeCarlo, E. H. Seawater transport and reaction in upper oceanic basaltic basement: Chemical data from continuous monitoring of sealed boreholes in a ridge flank environment. *Earth Planet. Sci. Lett.* **216,** 549–564 (2003).

3. Wheat, C. G. *et al.* Venting formation fluids from deep-sea boreholes in a ridge flank setting: ODP Sites 1025 and 1026. *Geochemistry, Geophys. Geosystems* **5,** (2004).

4. Wheat, C. G., Fisher, A. T., McManus, J., Hulme, S. M. & Orcutt, B. N. Cool seafloor hydrothermal springs reveal global geochemical fluxes. *Earth Planet. Sci. Lett.* **476,** 179–188 (2017).

5. Wheat, C. G., Hulme, S. M., Fisher, A. T., Orcutt, B. N. & Becker, K. Seawater recharge into oceanic crust: IODP Exp 327 Site U1363 Grizzly Bare outcrop. *Geochemistry, Geophys. Geosystems* **14,** 1957–1972 (2013).

6. Zimmermann, H. Tertiary seawater chemistry - Implications from primary fluid inclusions in marine halite. *Am. J. Sci.* **300,** 725–767 (2000).

7. Lowenstein, T. K., Timofeef, M. N., Brennan, S. T., Hardie, L. A. & Demicco, R. V. Oscillations in phaneroizoic seawater chemisrty: Evidence from fluid inclusions. *Science (80-.).* **294,** 1086–1088 (2001).

8. Horita, J., Zimmermann, H. & Holland, H. D. Chemical evolution of seawater during the Phanerozoic: Implications from the record of marine evaporites. *Geochim. Cosmochim. Acta* **66,** 3733–3756 (2002).

9. Brennan, S. T., Lowenstein, T. K. & Cendon, D. I. The major-ion composition of cenozoic seawater: the past 36 million years from fluid inclusions inmarine halite. *Am. J. Sci.* **313,** 713–775 (2013).

10. Berner, E. K. & Berner, R. A. *Global Environment: Water, Air and Geochemical Cycles.* (1996).

11. Elderfield, H. & Schultz, A. Mid-ocean ridge hydrothermal fluxes and the chemical composition of the ocean. *Annu. Rev. Earth Planet. Sci.* **24,** 191–224 (1996).

12. Arvidson, R. S., Mackenzie, F. T. & Guidry, M. MAGic: A Phanerozoic model for the geochemical cycling. *Am. J. Sci.* **306,** 135–190 (2006).

13. Vance, D., Teagle, D. A. H. & Foster, G. L. Variable Quaternary chemical weathering fluxes and imbalances in marine geochemical budgets. *Nature* **458,** 493–496 (2009).

14. Higgins, J. A. & Schrag, D. P. The Mg isotopic composition of Cenozoic seawater - evidence for a link between Mg-clays, seawater Mg/Ca, and climate. *Earth Planet. Sci. Lett.* **416,** 73–81 (2015).

15. Davis, A. C., Bickle, M. J. & Teagle, D. A. H. Imbalance in the oceanic strontium budget. *Earth Planet. Sci. Lett.* **211,** 173–187 (2003).

16. Mottl, M. J. in *Energy and Mass Transfer in Marine Hydrothermal Systems* 271–287 (2003).

17. Teagle, D. A. H., Bickle, M. J. & Alt, J. C. Recharge flux to ocean-ridge black smoker systems: A geochemical estimate from ODP Hole 504B. *Earth Planet. Sci. Lett.* **210,** 81–89 (2003).

18. Holland, H. D. Sea level, sediments, and the composition of seawater. *Am. J. Sci.* **305,** 220–239 (2005).

19. Fisher, A. & Wheat, C. G. Seamounts as Conduits for Massive Fluid, Heat, and Solute Fluxes on Ridge Flanks. *Oceanography* **23,** 74–87 (2010).

20. Gothmann, A. M., Stolarski, J., Adkins, J. F. & Higgins, J. A. A Cenozoic record of seawater Mg isotopes in well-preserved fossil corals. *Geology* **45,** 1039–1042 (2017).

21. Foster, G. L., Pogge Von Strandmann, P. A. E. & Rae, J. W. B. Boron and magnesium isotopic composition of seawater. *Geochemistry, Geophys. Geosystems* **11,** 1–10 (2010).

22. Ling, M. X. *et al.* Homogeneous magnesium isotopic composition of seawater: An excellent geostandard for Mg isotope analysis. *Rapid Commun. Mass Spectrom.* **25,** 2828–2836 (2011).

23. Tipper, E. T. *et al.* The magnesium isotope budget of the modern ocean: Constraints from riverine magnesium isotope ratios. *Earth Planet. Sci. Lett.* **250,** 241–253 (2006).

24. Higgins, J. A. & Schrag, D. P. Constraining magnesium cycling in marine sediments using magnesium isotopes. *Geochim. Cosmochim. Acta* **74,** 5039–5053 (2010).

25. An, Y. & Huang, F. A review of Mg isotope analytical methods by MC-ICP-MS. *J. Earth Sci.* **25,** 822–840 (2014).

26. Shalev, N. *et al.* Mg Isotope Interlaboratory Comparison of Reference Materials from Earth-Surface Low-Temperature Environments. *Geostand. Geoanalytical Res.* **42,** 205–221 (2018).
